# Role of the prescriber in supporting patients to discontinue benzodiazepines: a qualitative study

**DOI:** 10.3399/BJGP.2020.1062

**Published:** 2021-06-15

**Authors:** Erin Oldenhof, Timothy Mason, Jane Anderson-Wurf, Petra K Staiger

**Affiliations:** Faculty of Health, School of Psychology, Deakin University, Geelong; benzodiazepine counsellor, Reconnexion, a service of EACH, Melbourne.; School of Psychology, Deakin University, Geelong.; Reconnexion, a service of EACH, Melbourne.; Faculty of Health, School of Psychology, Deakin University, Geelong; deputy director, Centre for Drug Use, Addictive and Anti-social behaviour Research (CEDAAR), Deakin University, Geelong.

**Keywords:** benzodiazepines, deprescriptions, general practice, guidelines, patient-centred, perceptions, Z-drugs

## Abstract

**Background:**

Given the prevalence of long-term benzodiazepine (BZD) prescribing, increased monitoring through the implementation of prescription monitoring programmes (PMPs) may be the necessary impetus to promote BZD deprescribing. Despite evidence promoting the importance of patient-centred care, GPs have not been sufficiently supported to implement these principles through current deprescribing practice.

**Aim:**

To investigate patients’ perception of their prescriber’s influence on ceasing BZD use, including their willingness to take their advice, and to understand how a patient’s stage of change influences the barriers and facilitators they perceive to discontinuing BZDs.

**Design and setting:**

An online survey and qualitative interviews with 22 long-term users of BZD (≥6 months), aged 18–69 years, recruited from the general population in Victoria, Australia.

**Method:**

Two groups of users of BZD participated, one in the process of reducing their BZD and one not reducing, and were categorised according to their stage of change. Data underwent thematic analysis to identify barriers and facilitators to reducing BZDs both at the patient level and the prescriber level.

**Results:**

BZD patients’ perceptions of the prescriber influence were characterised by prescribing behaviours, treatment approach, and attitude. Barriers and facilitators to reducing their BZD were mapped against their stage of change. Irrespective of their stage of change, participants reported they would be willing to try reducing their BZD if they trusted their prescriber.

**Conclusion:**

This study illustrates that, with a few key strategies at each step of the deprescribing conversation, GPs are well positioned to tackle the issue of long-term BZD use in a manner that is patient centred.

## INTRODUCTION

Benzodiazepines (BZDs) are widely prescribed in the management of anxiety and insomnia, as well as for their muscle relaxant and anticonvulsant properties in conditions such as restless legs syndrome and epilepsy. However, guidelines call for short-term use (that is, ≤4 weeks),^1^^–^^3^ as BZDs taken regularly beyond this time can lead to iatrogenic dependence, emotional blunting and depression, cognitive deficits, motor vehicle accidents, falls in older adults, and mortality.^4^^–^^8^ Despite awareness of these harms, a significant disparity exists between evidence and practice as long-term BZD prescribing is commonly reported.^9^^–^^11^ A more recent concern is the rising number of BZD-related overdose deaths,^12^^–^^14^ which has led some governments to increase their regulation through the implementation of prescription monitoring programmes (PMPs). By centralising prescribing data, PMPs allow prescribers (that is, GPs) and pharmacists to more effectively monitor and manage BZDs and other high-risk medications. Evidence shows that PMPs do reduce the prescribing of monitored medications, but have also given rise to unintended consequences.^15^^–^^16^

While PMPs provide GPs with new information about patient risk, they offer little guidance on how to manage this risk, so GPs are more likely to respond in a risk-averse and reflexive manner (see Oldenhof and others^15^ for discussion), leading many patients to have their BZD prescription ceased inappropriately (that is, prescription abruptly terminated without exploration of indicated use).^17^^–^^18^ This new prescribing dilemma has seen many patients who take their BZD appropriately exposed to avoidable harms, as having their BZD reduced too rapidly (or cold turkey) can cause acute withdrawal (including seizures).^19^ It is critical, therefore, that GPs are equipped with sufficient skills and knowledge to safely deprescribe and start conversations to collaboratively address the risks identified by PMPs to improve patient outcomes.

Despite acknowledging the need for better management of BZD harm,^20^^–^^21^ it appears GPs struggle with the competing demands of providing patients with comfort and relief, and the reality of deprescribing interventions that can be challenging and uncomfortable.^20^^,^^22^ As a result, a GP’s attitude, skills, and knowledge regarding deprescribing influences the uptake and implementation of these interventions,^21^^,^^23^^–^^24^ where hesitation stems from insufficient experience and training rather than a lack of desire to address BZD harm.^22^^,^^25^ A recent review further emphasised the significance of this gap, showing that GP skills and knowledge were central components of patient-centred care, and the patient centredness of deprescribing interventions underpinned their efficacy.^26^

**Table:** How this fits in

The introduction of prescription monitoring programmes (PMPs) has highlighted the need for increased support and guidance to ensure GPs improve on current deprescribing practice. This study revealed the importance of GPs understanding how to empower patients through patient-centred care, to foster a willingness to try reducing, build motivation, and promote confidence in the patients’ ability to reduce. By understanding the patient experience with their prescriber involved in long-term BZD use, this study advances current knowledge of the ‘patient-centredness’ of deprescribing interventions and offers GPs clearer guidance on how to deliver these strategies effectively.

Though widely acknowledged that the principles of ‘patient-centred care’ should guide deprescribing conversations,^27^ this ubiquitous concept in health care has not been operationalised in a way that GPs can easily adhere to when deprescribing BZDs. Without sufficient guidance on *how* to navigate these conversations, it is unsurprising that GPs are often reluctant to initiate this process, as it is also perceived to be a demanding and thankless task.^22^ Patient-centred principles for deprescribing BZDs must be developed, beginning with a thorough understanding of the patients’ views and needs in relation to ceasing long-term BZD use. Examining patients’ experiences within the context of long-term BZD prescribing will also reveal important information around their expectations and attitude towards their prescriber. This will include why they might follow recommendations to reduce their BZD and offer insight into which GP characteristics (that is, skills and knowledge) facilitate the deprescribing process.

Previous investigation into the patient-level influences on ceasing long-term BZD has identified a range of barriers and facilitators to reducing BZDs.^28^ However, this body of research tends to reflect the experiences of older adults or other specific populations (that is, high-dose dependence),^26^^,^^29^ and is yet to explore how barriers and facilitators might differ depending on the patients’ readiness to change. In other words, the deprescribing conversation is likely very different with someone who has never considered reducing than with a patient who raises the concern themselves. Although the transtheoretical model (TTM; that is, ‘stages of change’) is recommended to guide deprescribing,^30^ at present only one study has validated the efficacy of BZD interventions when aligned with the patients’ stage of change.^31^ Broader consideration is therefore required to understand how patient barriers and facilitators vary over time according to their level of motivation and capacity for change. Evidence from alcohol research shows that GP strategies informed by stages of change are highly effective and time efficient, where a 5-minute conversation can influence alcohol use.^32^^–^^33^

This study marries a patient focus with a theoretical lens (TTM) to extend available evidence and explore the experiences of long-term BZD users within the general population. The primary aim was to investigate patients’ perception of their relationship with their GP, including what influences their willingness to accept advice and to understand the impact of prescriber characteristics and communication on the deprescribing process. This study also aimed to capture the views of individuals who are at different stages of change, in order to understand how the barriers and facilitators to discontinuing BZDs change over time.

## METHOD

### Participants

Two groups of long-term BZD users (defined as daily/near-daily use for ≥6 months) were recruited, one that was reducing their BZD (*n* = 11) and one that was not reducing (*n* = 11). Groups were characterised according to their stage of change;^34^ where those reducing were identified to be in ‘preparation’ if they intended to take action within a month, in ‘action’ if they had been reducing for <6 months, and in ‘maintenance’ if they had been reducing for >6 months. Those not reducing were characterised either as ‘precontemplation’ if they had no intention to reduce in the next 6 months, and ‘contemplation’ if they were thinking about reducing in the next 6 months and considering the risk–reward of ongoing BZD use.

Purposeful sampling and saturation principles were employed in recruitment, which occurred online via social media and also through a benzodiazepine specialist support service. Sample size was determined by saturation of codes and meaning, confirmed once no new codes or insights were identified regarding the research questions.^35^ Participants were informed that the goal of the study was to gain a greater understanding of the experiences of individuals prescribed BZDs. The relevant University Human Research Ethics Committee (DUHREC) approved the study. All participants gave written consent before participation and again after their interview to approve of their transcript, and received a gift voucher in compensation for their time.

### Data collection

In addition to the semi-structured interviews, participants completed a brief online questionnaire to gather demographic data, historic and current BZD use, and screens for mental and physical health. The latter enabled a summary of the clinical characteristics of the sample, and included the Patient Health Questionnaire-9^36^ (PHQ-9) to assess depressive symptoms, Generalised Anxiety Disorder-7^37^ (GAD-7) to assess symptoms of anxiety, the Health Survey Short-Form-8^38^ (SF-8) to assess physical health, and the Severity of Dependence Scale^39^ (SDS) to assess the level of BZD dependence. The interview guide was piloted before use, and initially invited participants to share their story about how they came to be prescribed a BZD, as well as exploring the advantages and disadvantages of taking their medication. This was followed by open-ended questions to assess the perceived barriers and enablers to discontinuing, and their relationship with their prescriber. Interviews were conducted by the lead researcher, a clinician experienced in clinical interviews and facilitating groups. Interviews were held one-on-one at university offices or via telephone and lasted between 19–57 minutes (mean = 34 minutes). Field notes and a reflective journal were kept. Interviews were audio-recorded and transcribed, with all identifiable data redacted from transcripts. Participants reviewed their transcripts before data analysis, with the opportunity to amend, comment on, and approve their transcript.

### Data analysis

All quantitative data analyses for descriptive statistics were conducted in SPSS (version 25) and qualitative analyses were performed using QSR NVivo (version 12). A phenomenologically informed approach to thematic analysis was chosen to generate patterns of meaning from the experiences, beliefs, and opinions of long-term BZD users. Codes were generated using an inductive approach, meaning they were derived from the data, and reflexive thematic analysis was carried out according to the guidelines by Braun and Clarke.^40^ Two researchers independently read all transcripts and coded three transcripts in parallel. Coders then met to discuss and review themes through an iterative process that saw several themes either merged or subsumed into subthemes. When discrepancies could not be resolved, they were reviewed in consultation with the team to achieve consensus. The consolidated criteria for reporting qualitative research (COREQ) guided reporting of the results.^41^

## RESULTS

### Quantitative results

#### Participants

Of the 36 participants who agreed to participate, 22 continued through to complete the interviews before saturation was met (three were non-contactable, two cancelled interviews, three no longer met eligibility, and six remained on the waitlist). Participant characteristics are outlined in Table 1. The mean daily dose equivalency to diazepam was 11.5 mg (± 6.1 mg), and nearly half (45.5%) of participants were prescribed a second BZD (or Z-drug), which was most commonly a hypnotic. Over three-quarters (77.3%) were prescribed another psychotropic medication, with the most common being a selective serotonin reuptake inhibitor.

### Qualitative results

Qualitative findings are presented to first outline the prescriber influence on reducing long-term BZD use followed by the patient influence. Each section offers a figure summarising the barriers and facilitators, which are then explained in greater depth (see Supplementary Table S1 for coding frame with exemplar quotes).

#### Prescriber influence

**Figure 1.**
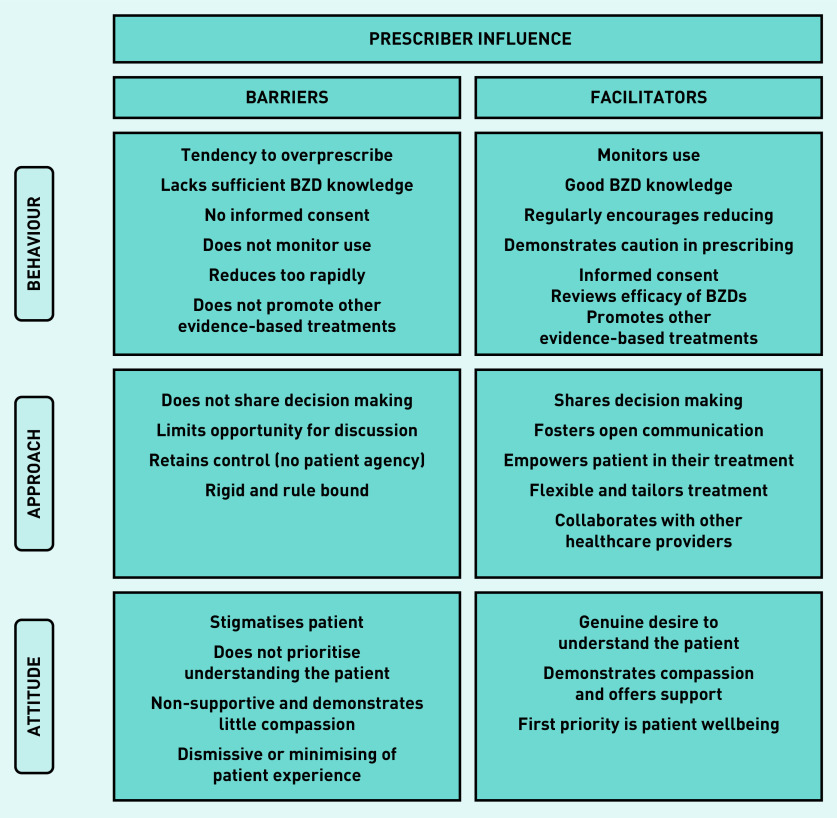
*Overview of the prescriber influence perceived to act as barriers or facilitators to reducing long-term use of BZDs. BZD = benzodiazepine.*

The participants’ perception of the prescriber influence on reducing their BZD were characterised into three broad categories (Figure 1). Barriers and facilitators are listed from the most to least common. Participant experiences of their prescriber reflected a broad divide between those in the process of reducing (that is, preparation, action, and maintenance) and those not currently reducing (precontemplation and contemplation), so themes are discussed to compare and contrast the experiences of these two groups.

#### Prescriber barriers

a) Prescribing behaviours: the majority of participants reducing, and a few not reducing, perceived issues with prescribers’ tendency to overprescribe and lack of sufficient knowledge about BZDs. Participants indicated that these behaviours led to their current dependent state, underpinned by a relaxed attitude towards their medication, inadequate awareness of harms, and little exploration of other evidence-based treatment options. Several participants described this as lacking a duty of care and expressed a generalised frustration towards the medical profession:

*‘… now at no stage did the GP say look we have to be careful ‘cause these are addictive, or you’ll get dependent on them.’*

(Participant [P]26, Action)

b) Prescriber treatment approach: more of those reducing than not expressed frustration or disappointment about behaviours that undermined their agency in their treatment, largely owing to limited consultation in decision making. Some described their prescriber adopting a one size fits all approach where they maintained control and authority in the relationship.

c) Prescriber attitude: common among reducers was the report of feeling stigmatised by their prescriber, that there was little effort to understand them, and that the level of support/compassion was insufficient. This meant participants felt unable to talk openly about their BZDs, and some interpreted the prescriber attitude as being dismissive or even punitive:

*‘And I said, “why did you put me on that, why did you tell me that it’s safe to use and it’s not addictive as long as you take it as prescribed?” I said, “I read all this on the internet, and I spoke to people”, and he said, “oh* […] *that’s all poppycock I wouldn’t listen to a word they say”, so* […] *just dismissed it out of hand.’*

(P10, Maintenance)

#### Prescriber facilitators

a) Prescribing behaviours: participants consistently spoke about prescriber behaviours described as a ‘duty of care’, consisting of satisfactory knowledge about BZDs, diligence in monitoring use, and regular suggestion of reducing. Participants indicated this conveyed confidence that their prescriber was supervising their use and promoted a feeling of safety. It also illustrated BZDs were a potentially harmful medication, so participants were conscious of not overrelying on their BZD:

‘ *… with my other medications he’ll give me repeats, but with my benzodiazepines he won’t, and he’ll monitor how often he prescribes them to keep me safe.’*

(P33, Precontemplation)

b) Prescriber treatment approach: the importance of being actively involved in their treatment was expressed equally by both groups, characterised by participating in decision making and voicing their opinion. Notable features of this prescriber approach were flexibility and collaboration with other healthcare providers (HCPs), and participants reporting a strong overall alliance with their prescriber:

*‘I think that if I was presenting more frequently* […] *she would have conversation with me about why* […] *because I don’t think she would want me to be taking more than what is prescribed or what is necessary, and she knows I don’t want to do that either.’*

(P17, Precontemplation)

c) Prescriber attitude: again, groups uniformly reported the importance of their prescriber making the effort to really understand them, sometimes meaning they went beyond their better judgement to support the patient (that is, to continue prescribing). Participants felt their prescriber demonstrated compassion and that their wellbeing was the prescribers’ first priority:

*‘If I take a double dose, then I say to her “I’ve had to double up”, she understands. I mean, because she has studied up on it* [medical condition] *, and she’s said to me “I’m really proud of you, what you’re taking” the low doses I’m taking of everything, compared to what she has read that others are taking. So, that made me feel good.’*

(P30, Precontemplation)

#### Patient influence

**Figure 2.**
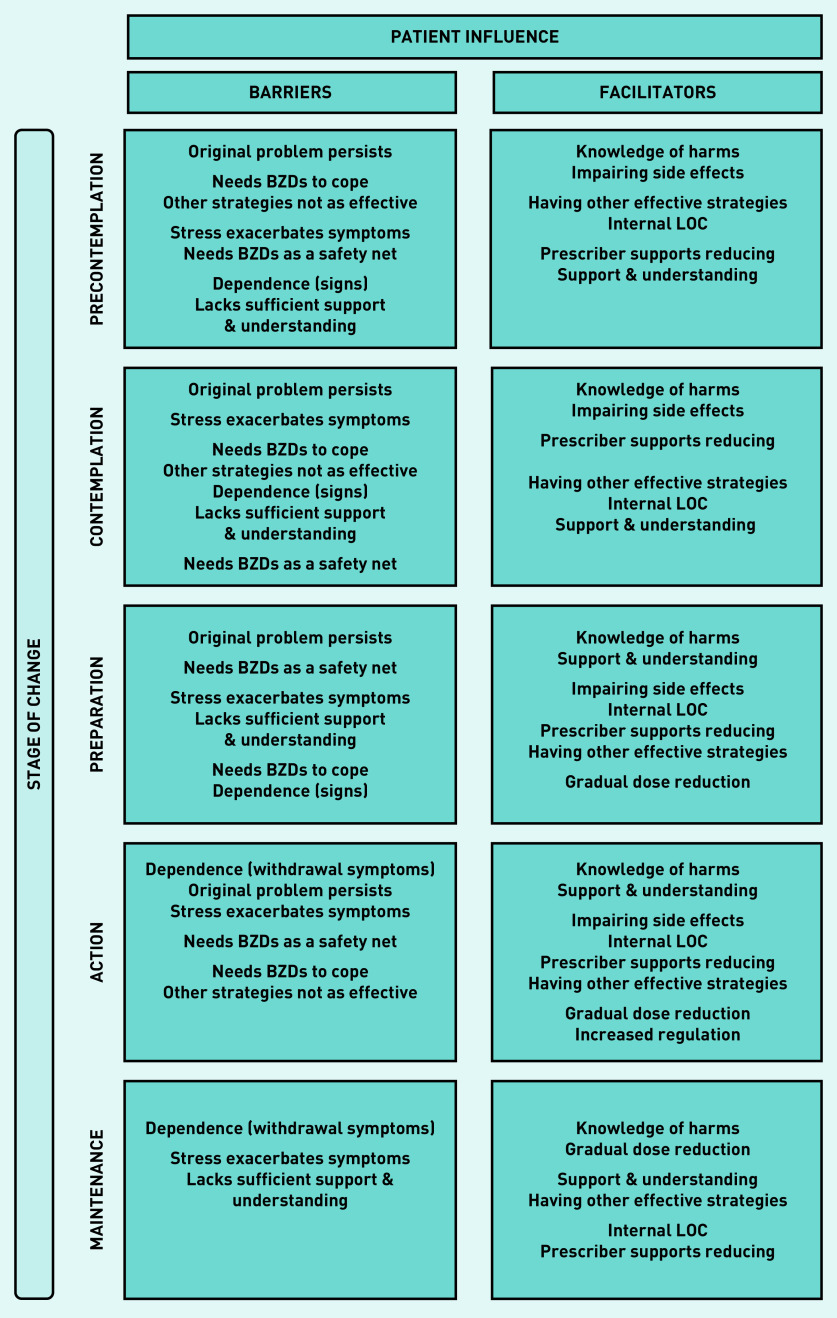
*Overview of patient influences that act as barriers or facilitators to reducing long-term use of BZDs. BZD = benzodiazepine. LOC = locus of control.*

An overview of the major themes identified as patient barriers and facilitators to reducing BZD use are displayed in Figure 2. These are presented in terms of the participants’ stage of change, highlighting the prominent barriers and facilitators at each stage to demonstrate how these experiences vary over time. Themes within each category are listed from the most to least commonly reported by participants.

#### Participants in precontemplation and contemplation stages

a) Barriers to reducing: for those not ready to reduce the most common barrier reflected a range of conditions and symptom severity. In some, the underlying condition was physical (for example, epilepsy) meaning they had little control over the need for their medication, but, for most, it was the impairing symptoms of their psychological condition (for example, post-traumatic stress disorder) that could not be managed by psychological intervention alone, and explained the need for the BZD to cope. A smaller number reported more generalised symptoms and were not trying to replace their BZD with other interventions. Participants also reported signs of dependence perpetuated BZD use, suggesting the primary benefit was either psychological relief or holding back withdrawal. The main contrast between these two stages was the greater focus on the impacts of stress in contemplators:

‘ *… it’s probably got to the point where it almost isn’t working as it should. Umm* […] *psychological thing, that you think, well if I don’t take it, I know I’m not going to sleep, and if I do take it, I’ll get a few hours.’*

(P29, Precontemplation)

b) Facilitators to reducing: although enablers did not outweigh the barriers for these two groups, knowing the harms associated with long-term use and experiencing impairing side effects did promote a more cautious attitude, with several participants limiting use where possible. Those in precontemplation described alternative coping strategies helping reduce their need for the BZD, whereas those in contemplation noted the prescribers’ support promoted a belief that they could reduce.

When thinking about reducing in the future, participants in both stages recognised the need to change their beliefs and attitude towards their medication (that is, internal locus of control [LOC]), but those in precontemplation were more likely to indicate they needed an alternative medication to replace their BZD:

*‘I’ve noticed that if I rely on having more than say two half tablets of diazepam in a day, I tend to be a bit too zonked out the following day. So, I try to limit myself to, say a half in the morning or a half at lunch, ideally less if I can, because of the work that I do* […] *I sort of need to be sharp.’*

(P18, Contemplation)

#### Participants in preparation stage

a) Barriers to reducing: participants in the preparation stage identified a greater number of pressing barriers than any other stage, suggesting that, when taking steps towards reducing, barriers become much more salient. This group were tentative about committing to permanent changes regarding their medication, and most needed access to their BZD assured, even if they planned to stop taking them.

b) Facilitators to reducing: facilitators for preparation and action stages were equal, so are discussed together.

#### Participants in action stage

a) Barriers to reducing: at this stage, withdrawal symptoms were the most significant barrier, which were exacerbated by life stressors. Participants were often unsure if these symptoms were their original problem or withdrawal related. In contrast to the precontemplation and contemplation stages, the reason these participants needed BZDs to cope was not just to improve functioning but also to make life easier in general and symptoms more tolerable:

*‘Yeah, it’s just the withdrawal, not only do I get the physical symptoms, like cold or flu, gastrointestinal problems, lumped on top of that I get high anxiety again and my thoughts race, I mean that’s what it seems like unless it’s still my condition.’*

(P34, Action)

b) Facilitators to reducing: participants in preparation and action stages placed equal significance not only on knowing the harms but also on the need for additional support and understanding to reduce, a sign they were taking steps to aid the process. They also described a growing sense of confidence and control about reducing — underpinned by an internal LOC — which was the benefit of the prescribers’ ongoing encouragement and a gradual dose reduction. Of note, this group uniquely reported the influence of increased regulation on prompting their decision to discontinue use:

*‘It was never my intention to get it like, illegally or through you know, manipulation or anything like that. And when it started getting harder and hard to get, I just thought, ‘’well screw it, I’m going off it, cause I don’t want to feel that way’’.’*

(P34, Action)

#### Participants in maintenance stage

a) Barriers to reducing: in the maintenance stage, withdrawal symptoms remained the primary barrier to coming off the BZD; and, again, the impact of stress on these symptoms was also perceived to hinder the process. In addition, insufficient support from HCPs and a lack of understanding from family/friends further affected this group’s ability to reduce.

b) Facilitators to reducing: as the group with the longest experience reducing, beyond knowing the harms, a gradual dose reduction was now considered the most important facilitator. Nearly all who spoke about a gradual taper also expressed agency over this process, where the reduction rate was guided by them and how well they were coping:

*‘Well going off ‘em slowly, each dose drop adjusts into my system. I’ll judge on how I’m feeling with each taper, and adjust it from there, whether I stay on that extra dose for a bit longer, or I’m feeling OK and drop the next milligram and so forth.’*

(P23, Maintenance)

## DISCUSSION

### Summary

Exploring the prescriber influence revealed that a participant’s willingness to take their prescriber’s advice was determined by their evaluation of whether the prescriber’s behaviour, approach, and attitude to treatment led them to trust their prescriber. The most significant influences on trust were the prescriber having a genuine desire to understand the patient, being knowledgeable about BZDs, open communication, shared decision making, and, to a lesser degree, the duration of the relationship. It is noteworthy that participants in precontemplation and contemplation stages who trusted their prescriber also indicated that they would attempt reducing their BZD if their prescriber recommended it. Participants who indicated they lacked trust in their prescriber also indicated that the prescriber’s approach (that is, retaining control and not encouraging open communication), as well as their behaviour (that is, lack of knowledge about BZDs and lack of monitoring), influenced this evaluation of trust.

The barriers and facilitators to reducing long-term use of BZDs in participants from the general population were similar at each stage of change, but their level of importance varied. The key barriers were identified as the ‘original problem persists’, ‘stress exacerbates symptoms’, ‘dependence’ (that is, either signs of dependence or onset of withdrawal symptoms), ‘need BZDs to cope’ with everyday life, ‘other strategies not as effective’, ‘lack sufficient support and understanding’, and ‘need BZD as a safety net’. The facilitators to discontinuing were ‘knowledge of harms’ associated with long-term use, experiencing ‘impairing side effects’, having ‘other effective strategies’, receiving ‘support and understanding’ to address the underlying issue(s), an ‘internal LOC’, ‘prescriber supports reducing’, a ‘gradual dose reduction’, and ‘increased regulation’.

### Strengths and limitations

To the authors’ knowledge, this is the first study to explore patient influences on reducing long-term BZD use across a spectrum of change, while also identifying how the influence of the prescriber can either support or hinder this process. The data have good validity as they reflect a broad range of ages, genders, and experiences relating to BZD use, and a range of presentations with half of the sample experiencing mental health comorbidities (that is, 45.5% met clinical cut-offs on both PHQ-9 and GAD-7). However, there are some drawbacks to the generalisability and representativeness of the sample, in that most participants were from a metropolitan area, and only three were from diverse ethnic backgrounds. Also, recruitment via social media and a specialist service may have inherently ruled out a portion of the population from the sampling pool. Last, the focus of this paper was on improving deprescribing practice and did not address the important issue of identifying effective treatments that might supplant the need for the BZD.

### Comparison with existing literature

One previous study hypothesised that BZD cessation was more successful when led by the regular prescriber rather than by another HCP, owing to the trusting and understanding relationship.^42^ Present findings supported this premise and showed that trust was the value judgement that facilitated a patient’s willingness to adhere to their prescriber’s advice, even when this conflicted with their own perspective. This suggests that the patient–prescriber relationship itself, comprising the quality and content of transactions over time, can be a facilitator of change in deprescribing long-term use of BZDs.

Patient barriers and facilitators identified in this study were consistent with the literature^28^^,^^43^ but go beyond current understanding, illustrating how these influences vary according to the patients’ stage of change. Previous research shows that, when used long term, BZDs often become necessary to cope and provide not just symptom relief, but also offer comfort, security, and a buffer to life stressors.^44^^–^^45^ This study’s findings suggest this need is prevalent only in precontemplation or contemplation stages and diminishes as the patient begins planning and actively reducing their BZD. Similarly, several studies have shown a patient’s lack of knowledge about harms perpetuates long-term use;^46^^–^^48^ however, here it was shown that knowledge was also the primary facilitator for reducing. This highlights how instrumental patient education is both to decreasing the risk for and facilitating the cessation of long-term use.

Although limited, some studies have emphasised the role of self-efficacy in facilitating a patient’s capacity to reduce, and how internalising the LOC increases a patient’s confidence in their ability to reduce.^31^^,^^49^ Applying the TTM, it was revealed that supporting these internal resources is crucial at the preparation stage, when participants perceived the greatest number of pressing barriers to their reduction. This is the time when receiving additional support to address the underlying issue becomes a key facilitator, a process expected to build patient confidence (self-efficacy) and a sense of control (internal LOC) in managing their symptoms in order to reduce their BZD.

Last, the potential for PMPs to be an impetus for deprescribing BZDs was evident in the current sample, as a small number of participants reported increased government regulation prompted their decision to discontinue use. Although able to access support to guide their cessation, these individuals experienced either stigma or reproach in how the prescriber communicated these new regulations, reiterating the need for patient-centred principles to be adopted alongside the implementation of PMPs.^15^

### Implications for practice

This study has distilled priorities for GPs with recommendations that consider both prescriber and patient influence on reducing long-term BZD use. As illustrated in Box 1, adopting a stage-based approach clarifies specific tasks for GPs and equips them with the knowledge of what to expect (that is, patient barriers) and strategies to overcome these by targeting patient facilitators.

**Box 1.** A stage-based approach to deprescribing BZDs

	**Patient Influence**		**Prescriber Influence**	
**Stage of change**	**Main barriers**	**Main facilitators**	**Facilitatory behaviours**	**Facilitatory approach**
**Precontemplation**				
(No intention to reduce in the next 6 months)	Original problem persists	Knowledge of harms Experiencing impairing side effects	→ Inform patient of potential harms → Monitor use and review efficacy → Regularly encourage reducing	→ Open conversation around BZD harms and side effects → Cautious approach to prescribing (that is, rational prescribing)
**Contemplation**				
(Considering reducing in the next 6 months)	Original problem persists Stress exacerbates symptoms	Knowledge of harms Experiencing impairing side effects	→ Monitor use and review efficacy → Promote other evidence-based treatments → Support reducing	→ Curious approach to understand patient experience of harms and benefits → Support patient to choose change
**Preparation**				
(Actively planning to reduce in the next month)	Original problem persists Need BZDs as a safety net Stress exacerbates symptoms Lack sufficient support and understanding	Knowledge of harms Professional support and understanding from family and/or friends	→ Generate a GDR plan → Psychoeducation around withdrawal → Coordination of care → Explore informal supports	→ Explore the ‘role’ that BZDs play in patient’s life → Collaborative approach to exploring how patient will eventually replace BZD (that is, internal and external coping strategies)
**Action**				
(Reducing for <6 months)	Withdrawal symptoms Original problem persists Stress exacerbates symptoms	Knowledge of harms Professional support and understanding from family and/or friends	→ Review and adjust GDR with patient as required → Ensure patient is sufficiently supported (formal and informal support)	→ Offer ongoing encouragement → Supportive approach to normalise worries about ability to reduce
**Maintenance**				
(Reducing for >6 months)	Withdrawal symptoms	Knowledge of harms Gradual dose reduction	→ Review and adjust GDR with patient as required → Consider if other treatments are required	→ Explore efficacy of other interventions → Collaborative and flexible approach for duration of reduction

*BZD = benzodiazepine. GDR = gradual dose reduction.*

**Table 1.** Participant characteristics

**Characteristic**	**Total (*N*= 22)**	
**Age, mean ± SD**	42.6 ± 15.9	

**Identified gender,** ***n* (%)**		
Female	12 (54.5)	
Male	7 (31.8)	
Non-binary	3 (13.6)	

**Employment status,** ***n* (%)**		
Student (casual)	2 (9.1)	
Part time	2 (9.1)	
Full time	8 (36.4)	
Retired	3 (13.6)	
Unemployed/disability pension	4 (18.2)/3 (13.6)	

**BZD/Z-drug**	*Primary*	*Secondary*

Alprazolam	1	—
Clonazepam	2	—
Diazepam	14	1
Lorazepam	2	1
Nitrazepam	1	—
Oxazepam	1	1
Temazepam	—	3
Zopiclone/Zolpidem	1	4

**Duration of use, mean ± SD**	10.4 years ± 8.6	
1–2 years	7 (31.8%)	
5–10 years	6 (27.3%)	
11–≥20 years	9 (40.9%)	

**Reason prescribed**	*Primary*	*Secondary*

Anxiety disorder (including PTSD)	16	3
Insomnia	2	6
Medical condition	2	—
Substance withdrawal	2	—

**SDS scores (mean ± SD)**	5.0 ± 3.7	
**PHQ-9 scores (mean ± SD)**	11.4 ± 7.0	
**GAD-7 scores (mean ± SD)**	10.1 ± 5.0	
**SF-8 scores (mean ± SD)**	25.5 ± 5.3	

*BZD = benzodiazepine. GAD-7 = Generalised Anxiety Disorder-7. PHQ-9 = Patient Health Questionnaire-9. PTSD = post-traumatic stress disorder. SD = standard deviation. SDS = Severity of Dependence Scale. SF-8 = Health Survey Short-Form-8.*

The goal to achieve a shared understanding of the problem and an agreed-upon treatment approach, through open communication and shared decision making, will promote patients’ trust in the prescriber and a willingness to attempt reducing. This also requires a shared understanding that other interventions may be more suitable for treating persisting issues and acknowledging the more limited role of BZDs. By offering clear advice on how and what to communicate depending on the patients’ stage of change and drawing attention to the importance of the patient–prescriber relationship, present findings advance guidelines for a patient-centred approach to deprescribing BZDs.

It must be clarified, however, that not all instances of long-term BZD use reflect inappropriate prescribing. Significant complexities can underpin long-term use, and these require extended treatment to ensure they are carefully explored and addressed. However, where rational prescribing is necessary, patients must be informed of the full range of potential harms, treatment should be regularly reviewed, and a plan should be in place for ceasing the BZD.^2^^,^^50^

Recent calls for more realistic approaches to deprescribing highlight the importance of shared decision making, collaborative treatment exploration, and understanding the person as a 'whole', while incorporating evidence-based guidelines.^51^ Despite increased knowledge of BZD-related harms, they are yet to be adequately addressed in primary care, and the rise of PMPs may only increase patient harm to those already involved in long-term use. For many, longterm BZD use is perpetuated by a lack of knowledge about the potential harms. Therefore, raising patient awareness and engaging them more actively in their treatment are critical steps to facilitate discontinuation.

This study illustrates that with a few key strategies at each step of the deprescribing conversation, and where patients experience genuine concern for their wellbeing and are actively consulted throughout the process, prescribers are well positioned to tackle the issue of long-term BZD use. By adopting this approach, GPs will be better prepared to respond to patients identified as being at risk by PMPs, where deprescribing may be required, and to ensure they are patient centred throughout the entire process.
